# (*2R*)-8-Benzyl-2-[(*S*)-hy­droxy(phen­yl)meth­yl]-8-aza­bicyclo­[3.2.1]octan-3-one

**DOI:** 10.1107/S1600536811053190

**Published:** 2011-12-17

**Authors:** Krzysztof Brzezinski, Ryszard Lazny, Michal Sienkiewicz, Sławomir Wojtulewski, Zbigniew Dauter

**Affiliations:** a Synchrotron Radiation Research Section, MCL, National Cancer Institute, Argonne National Laboratory, Biosciences Division, Bldg. 202, Argonne, IL 60439, USA; b Institute of Chemistry, University of Bialystok, Hurtowa 1, 15-399 Bialystok, Poland

## Abstract

The crystal of the title compound, C_21_H_23_NO_2_, was chosen from a conglomerate formed by a racemic mixture. An intra­molecular hydrogen bond is formed between hy­droxy group and heterocyclic N atom of the aza­bicyclo­[3.2.1]octan-3-one system. The crystal structure is stabilized by C—H⋯O inter­actions between aliphatic C—H groups and the carbonyl O atom. For the title chiral crystal, the highly redundant and accurate diffraction data set collected with low energy copper radiation gave a Flack parameter of 0.12 (18) for anomalous scattering effects originating from O atoms.

## Related literature

For recent background literature on the chemistry of related tropane-derived aldols and their applications, including stereoselective syntheses of bioactive alkaloids, see: Lazny *et al.* (2011 ▶); Sienkiewicz *et al.* (2009 ▶) and references cited therein. For stereoselective syntheses of related nortropinone aldols, see: Lazny *et al.* (2001 ▶); Lazny & Nodzewska (2003 ▶). For a representative review of the biological activity of tropane derivatives, see: Singh (2000 ▶).
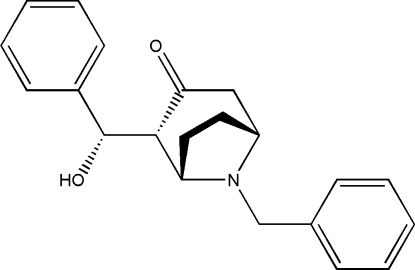


## Experimental

### 

#### Crystal data

C_21_H_23_NO_2_
*M* *_r_* = 321.40
Orthorhombic, 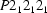

*a* = 5.9354 (1) Å
*b* = 13.3091 (2) Å
*c* = 22.1511 (3) Å
*V* = 1749.82 (5) Å^3^
*Z* = 4
Cu *K*α radiation
μ = 0.61 mm^−1^
*T* = 100 K
0.65 × 0.25 × 0.19 mm

#### Data collection

Oxford Diffraction SuperNova Dual diffractometer
Absorption correction: analytical (*CrysAlis PRO*; Agilent, 2011 ▶) *T*_min_ = 0.75, *T*_max_ = 0.89
32829 measured reflections
3323 independent reflections
3276 reflections with *I* > 2σ(*I*)
*R*_int_ = 0.026

#### Refinement

*R*[*F* ^2^ > 2σ(*F* ^2^)] = 0.027
*wR*(*F* ^2^) = 0.070
*S* = 1.18
3323 reflections
218 parameters
H-atom parameters constrained
Δρ_max_ = 0.16 e Å^−3^
Δρ_min_ = −0.16 e Å^−3^
Absolute structure: Flack (1983 ▶), 1257 Friedel pairs
Flack parameter: 0.12 (18)

### 

Data collection: *CrysAlis PRO* (Agilent, 2011 ▶); cell refinement: *CrysAlis PRO*; data reduction: *CrysAlis PRO*; program(s) used to solve structure: *SHELXD* (Sheldrick, 2008 ▶); program(s) used to refine structure: *SHELXL97* (Sheldrick, 2008 ▶); molecular graphics: *ORTEP-3* (Farrugia, 1997 ▶) and *pyMOL* (DeLano, 2002 ▶); software used to prepare material for publication: *SHELXL97*.

## Supplementary Material

Crystal structure: contains datablock(s) global, I. DOI: 10.1107/S1600536811053190/gk2429sup1.cif

Structure factors: contains datablock(s) I. DOI: 10.1107/S1600536811053190/gk2429Isup2.hkl

Supplementary material file. DOI: 10.1107/S1600536811053190/gk2429Isup3.cml

Additional supplementary materials:  crystallographic information; 3D view; checkCIF report

## Figures and Tables

**Table 1:** Hydrogen-bond geometry (Å, °)

*D*—H⋯*A*	*D*—H	H⋯*A*	*D*⋯*A*	*D*—H⋯*A*
O9—H9⋯N8	0.84	1.99	2.7280 (13)	146
C6—H6*B*⋯O3^i^	0.99	2.61	3.3414 (15)	131
C7—H7*A*⋯O3^i^	0.99	2.52	3.2954 (15)	135
C16—H16*A*⋯O3^i^	0.99	2.60	3.5846 (16)	173

Symmetry code: (i) 
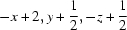
.

**Fig. 1.**
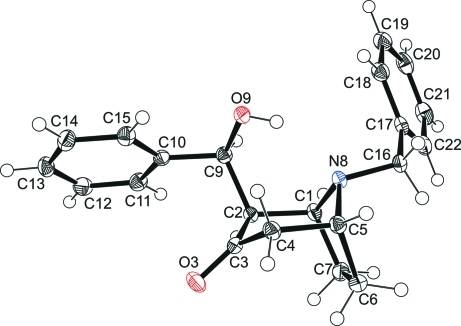
The molecular structure of the title compound. Displacement ellipsoids are drawn at the 50% probability level.

**Fig. 2.**
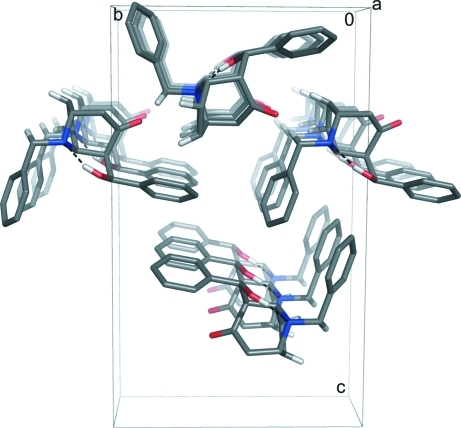
Crystal packing viewed along the a axis. Dashed lines represent hydrogen bonds. For clarity, only hydrogen atoms involved in the intra- and intermolecular interactions are shown.

## supplementary crystallographic information

### Comment

Tropane (8-methyl-8-azabicyclo[3.2.1]octane) and nortropane (8-azabicyclo[3.2.1]octane) are known scaffolds of numerous natural alkaloids, many of which demonstrate a range of biological activities. Many synthetic derivatives and unnatural analogues of tropane alkaloids have been synthesized and studied as potential agrochemically or pharmaceutically useful agents (Singh, 2000). Diastereomerically and enantimerically pure aldols of tropinone were used as key intermediates in stereoselective synthesis of unnatural enantiomer of cocaine (*ent*-cocaine), knightinol, alkaloid KD–B and ferrugine (Sienkiewicz *et al.*, 2009). Stereoselective syntheses of nortropinone aldols (Lazny & Nodzewska, 2003; Lazny *et al.*, 2001) is more complicated and remains a challenge. Therefore synthetically equivalent *N*-benzylnortropinone aldols may open a route to synthetic availability of nor-analogues of potential pharmaceutical importance. Knowledge of the structure and reactivity of the *N*-benzyl analogues of tropanes is also used for modeling reactivity of nortropanes anchored through nitrogen on commonly used solid-phase supports with benzyl derived linkers. The solid-phase immobilization and subsequent transformations are typically used in combinatorial approaches to preparation of libraries of potentially bioactive substances.

The studied *N*-benzyl compound was prepared by a procedure analogous to method known for *N*-methyl aldols. The synthetic procedure gave a racemic mixture, however homochiral crystals were formed spontaneously. An enantiomorphic crystal was picked at random.

The crystal structure of the title compound contains one molecule in the asymmetric unit (Fig. 1). The Flack parameter is equal to 0.12 (18) for the crystals containing (2*R*)-8-benzyl-2-[(*S*)-hydroxy(phenyl)methyl]-8-azabicyclo[3.2.1] octan-3-one enantiomer. The intramolecular hydrogen bond is formed between hydroxyl group and heterocyclic nitrogen atom from the azabicyclo[3.2.1]octan-3-one system. The carbonyl oxygen atom is located near equatorial hydrogen atoms of C6 and C7, as well as, the H16A atom. Intra- and intermolecular interactions are shown in Fig. 2 and summarized in Table 1.

### Experimental

A solution of n-butyllithium in hexane (2.4 *M*, 0.50 ml, 1.2 mmol) was added dropwise to a cooled (273 K) solution of diisopropylamine (0.168 ml, 1.2 mmol) in tetrahydrofuran (10 ml). The mixture was stirred for 30 min, then cooled to 195 K and a solution of *N*-benzylnortropinone (0.215 g, 1 mmol) in tetrahydrofuran (7 ml) was added dropwise. After stirring for 2 h, benzaldehyde (0.117 ml, 1.15 mmol) was added dropwise and the mixture was stirred for another 10 min. The reaction was quenched with saturated aq. NH_4_Cl (4 ml), allowed to warm to room temperature, and extracted with dichloromethane (3 × 10 ml). The combined extracts were dried over MgSO_4_ and concentrated to give the crude product. Crystallization from mixed solvent system hexane/dichloromethane gave the the major product (0.243 g, 75%) as white crystals [m.p. 372–377 K; *R*_f_ = 0.77 (10% methanol/dichloromethane); HR (MS-ESI): MNa+, found 344,1640, C_21_H_23_NNaO_2_ requires 344,1626; ^1^H NMR (CDCl_3_): 7.43–7.21 (m, 10H), 5.11 (d, *J* = 3 Hz, 1H), 3.73–3.65 (m, 3H), 3.58–3.57 (m, 1H), 2.82 (ddd, *J_1_* = 1.5 Hz, *J_2_* = 4.5 Hz, *J_3_* = 6 Hz, 1H), 2.45–2.44 (m, 1H), 2.36–2.32 (m, 3H), 1.70–1.66 (m, 2H)].

### Refinement

All hydrogen atoms were constrained to idealized positions with C—H distances fixed at 0.95–1.00 Å and O—H distances fixed at 0.84 Å and *U*_iso_(H) = 1.5*U*_eq_(C) for hydroxyl hydrogen atom and 1.2*U*_eq_(C) for others.

### Crystal data

**Table:**

C_21_H_23_NO_2_	*F*(000) = 688
*M**_r_* = 321.40	*D*_x_ = 1.220 Mg m^−^^3^
Orthorhombic, *P*2_1_2_1_2_1_	Cu *K*α radiation, λ = 1.54180 Å
Hall symbol: P 2ac 2ab	Cell parameters from 23874 reflections
*a* = 5.9354 (1) Å	θ = 3.3–73.6°
*b* = 13.3091 (2) Å	µ = 0.61 mm^−^^1^
*c* = 22.1511 (3) Å	*T* = 100 K
*V* = 1749.82 (5) Å^3^	Needle, colourless
*Z* = 4	0.65 × 0.25 × 0.19 mm

### Data collection

**Table:**

Oxford Diffraction SuperNova Dual diffractometer	3323 independent reflections
Radiation source: SuperNova (Cu) X-ray Source	3276 reflections with *I* > 2σ(*I*)
mirror	*R*_int_ = 0.026
Detector resolution: 10.4052 pixels mm^-1^	θ_max_ = 73.6°, θ_min_ = 3.9°
ω scans	*h* = −7→6
Absorption correction: analytical (*CrysAlis PRO*; Agilent, 2011)	*k* = 0→16
*T*_min_ = 0.75, *T*_max_ = 0.89	*l* = 0→27
32829 measured reflections	

### Refinement

**Table:**

Refinement on *F*^2^	Secondary atom site location: difference Fourier map
Least-squares matrix: full	Hydrogen site location: inferred from neighbouring sites
*R*[*F*^2^ > 2σ(*F*^2^)] = 0.027	H-atom parameters constrained
*wR*(*F*^2^) = 0.070	*w* = 1/[σ^2^(*F*_o_^2^) + (0.0283*P*)^2^ + 0.4297*P*] where *P* = (*F*_o_^2^ + 2*F*_c_^2^)/3
*S* = 1.18	(Δ/σ)_max_ < 0.001
3323 reflections	Δρ_max_ = 0.16 e Å^−^^3^
218 parameters	Δρ_min_ = −0.16 e Å^−^^3^
0 restraints	Absolute structure: Flack (1983), 1257 Friedel pairs
Primary atom site location: structure-invariant direct methods	Flack parameter: 0.12 (18)

### Special details

**Table:**

Geometry. All e.s.d.'s are estimated using the full covariance matrix. The cell e.s.d.'s are taken into account individually in the estimation of e.s.d.'s in distances, angles and torsion angles; correlations between e.s.d.'s in cell parameters are only used when they are defined by crystal symmetry.
Refinement. Refinement of *F*^2^ against all reflections. The weighted *R*-factor *wR* and goodness of fit *S* are based on *F*^2^, conventional *R*-factors *R* are based on *F*, with *F* set to zero for negative *F*^2^. The threshold expression of *F*^2^ > 2σ(*F*^2^) is used only for calculating *R*-factors *etc*. and is not relevant to the choice of reflections for refinement. *R*-factors based on *F*^2^ are statistically about twice as large as those based on *F*.

### Fractional atomic coordinates and isotropic or equivalent isotropic displacement parameters (Å^2^)

**Table:**

	*x*	*y*	*z*	*U*_iso_*/*U*_eq_	
C1	0.8760 (2)	0.63105 (8)	0.16890 (5)	0.0158 (2)	
H1	0.9172	0.6656	0.1304	0.019*	
C2	0.8530 (2)	0.51680 (8)	0.15913 (5)	0.0153 (2)	
H2	1.0031	0.4903	0.1462	0.018*	
C3	0.7921 (2)	0.46720 (9)	0.21973 (5)	0.0169 (3)	
O3	0.88275 (17)	0.38983 (7)	0.23623 (4)	0.0256 (2)	
C4	0.6185 (2)	0.52123 (8)	0.25898 (5)	0.0177 (3)	
H4A	0.6293	0.4967	0.3011	0.021*	
H4B	0.4649	0.5066	0.2439	0.021*	
C5	0.6612 (2)	0.63510 (8)	0.25734 (5)	0.0164 (2)	
H5	0.5476	0.6719	0.2823	0.020*	
C6	0.9079 (2)	0.65823 (10)	0.27939 (6)	0.0202 (3)	
H6A	0.9603	0.6067	0.3084	0.024*	
H6B	0.9166	0.7252	0.2987	0.024*	
C7	1.0516 (2)	0.65523 (9)	0.21957 (6)	0.0204 (3)	
H7A	1.1253	0.7208	0.2121	0.025*	
H7B	1.1686	0.6024	0.2218	0.025*	
N8	0.65350 (19)	0.66906 (7)	0.19242 (4)	0.0150 (2)	
O9	0.45290 (16)	0.52672 (6)	0.12196 (4)	0.0204 (2)	
H9	0.4614	0.5808	0.1414	0.031*	
C9	0.6768 (2)	0.49154 (9)	0.10786 (5)	0.0165 (3)	
H9C	0.7269	0.5263	0.0701	0.020*	
C10	0.6686 (2)	0.38004 (9)	0.09495 (5)	0.0166 (3)	
C11	0.8557 (3)	0.33441 (10)	0.06662 (5)	0.0210 (3)	
H11	0.9814	0.3742	0.0551	0.025*	
C12	0.8568 (3)	0.23166 (10)	0.05557 (6)	0.0238 (3)	
H12	0.9833	0.2009	0.0369	0.029*	
C13	0.6694 (3)	0.17393 (9)	0.07224 (6)	0.0241 (3)	
H13	0.6699	0.1035	0.0652	0.029*	
C14	0.4813 (3)	0.21910 (10)	0.09918 (6)	0.0226 (3)	
H14	0.3537	0.1794	0.1093	0.027*	
C15	0.4803 (2)	0.32179 (9)	0.11116 (5)	0.0196 (3)	
H15	0.3539	0.3522	0.1301	0.023*	
C16	0.6383 (2)	0.78046 (8)	0.19028 (5)	0.0171 (3)	
H16A	0.7639	0.8098	0.2138	0.020*	
H16B	0.4952	0.8022	0.2091	0.020*	
C17	0.6486 (2)	0.81981 (8)	0.12530 (6)	0.0176 (3)	
C18	0.4719 (3)	0.80051 (9)	0.08410 (6)	0.0212 (3)	
H18	0.3449	0.7626	0.0969	0.025*	
C19	0.4813 (3)	0.83679 (10)	0.02427 (6)	0.0252 (3)	
H19	0.3614	0.8234	−0.0030	0.030*	
C20	0.6690 (3)	0.89285 (10)	0.00518 (6)	0.0262 (3)	
H20	0.6763	0.9177	−0.0350	0.031*	
C21	0.8457 (3)	0.91194 (9)	0.04567 (6)	0.0265 (3)	
H21	0.9729	0.9494	0.0326	0.032*	
C22	0.8362 (3)	0.87590 (9)	0.10572 (6)	0.0227 (3)	
H22	0.9564	0.8895	0.1328	0.027*	

### Atomic displacement parameters (Å^2^)

**Table:**

	*U*^11^	*U*^22^	*U*^33^	*U*^12^	*U*^13^	*U*^23^
C1	0.0150 (6)	0.0132 (5)	0.0194 (6)	0.0009 (5)	0.0025 (5)	−0.0001 (4)
C2	0.0143 (6)	0.0129 (5)	0.0186 (5)	0.0017 (5)	−0.0010 (5)	−0.0008 (4)
C3	0.0174 (7)	0.0140 (5)	0.0192 (6)	−0.0017 (5)	−0.0041 (5)	−0.0006 (5)
O3	0.0324 (6)	0.0175 (4)	0.0269 (5)	0.0076 (4)	−0.0038 (4)	0.0032 (4)
C4	0.0197 (7)	0.0154 (5)	0.0178 (5)	0.0003 (5)	0.0000 (5)	0.0026 (4)
C5	0.0180 (7)	0.0157 (5)	0.0156 (5)	0.0014 (5)	0.0006 (5)	−0.0002 (4)
C6	0.0210 (8)	0.0187 (6)	0.0207 (6)	0.0000 (5)	−0.0046 (5)	−0.0004 (5)
C7	0.0147 (7)	0.0183 (6)	0.0283 (7)	0.0003 (5)	−0.0008 (5)	−0.0043 (5)
N8	0.0169 (6)	0.0120 (4)	0.0160 (5)	0.0020 (4)	0.0010 (4)	0.0004 (4)
O9	0.0178 (5)	0.0165 (4)	0.0269 (5)	0.0037 (3)	−0.0038 (4)	−0.0048 (4)
C9	0.0179 (7)	0.0157 (5)	0.0159 (5)	0.0005 (5)	0.0003 (5)	0.0004 (4)
C10	0.0207 (7)	0.0166 (5)	0.0126 (5)	0.0006 (5)	−0.0028 (5)	−0.0009 (4)
C11	0.0220 (7)	0.0223 (6)	0.0187 (6)	−0.0001 (5)	0.0002 (5)	−0.0029 (5)
C12	0.0260 (8)	0.0246 (6)	0.0209 (6)	0.0072 (6)	−0.0024 (6)	−0.0065 (5)
C13	0.0371 (9)	0.0155 (6)	0.0196 (6)	0.0019 (6)	−0.0052 (6)	−0.0030 (5)
C14	0.0309 (8)	0.0194 (6)	0.0175 (6)	−0.0055 (6)	−0.0018 (6)	0.0006 (5)
C15	0.0228 (7)	0.0197 (6)	0.0162 (5)	−0.0005 (5)	0.0008 (5)	−0.0012 (5)
C16	0.0188 (7)	0.0118 (5)	0.0206 (6)	0.0014 (5)	−0.0005 (5)	−0.0013 (4)
C17	0.0194 (7)	0.0110 (5)	0.0224 (6)	0.0034 (5)	0.0025 (6)	0.0002 (4)
C18	0.0228 (8)	0.0171 (6)	0.0238 (6)	0.0003 (5)	0.0003 (5)	0.0034 (5)
C19	0.0302 (8)	0.0218 (6)	0.0235 (6)	0.0036 (6)	−0.0033 (6)	0.0021 (5)
C20	0.0369 (9)	0.0192 (6)	0.0226 (6)	0.0066 (6)	0.0074 (6)	0.0049 (5)
C21	0.0261 (8)	0.0187 (6)	0.0347 (7)	0.0010 (6)	0.0113 (6)	0.0046 (5)
C22	0.0226 (8)	0.0153 (5)	0.0303 (7)	0.0009 (5)	0.0020 (6)	0.0007 (5)

### Geometric parameters (Å, °)

**Table:**

C1—N8	1.5071 (16)	C10—C15	1.4068 (19)
C1—C2	1.5419 (15)	C10—C11	1.4127 (19)
C1—C7	1.5648 (18)	C11—C12	1.3893 (18)
C1—H1	1.0000	C11—H11	0.9500
C2—C3	1.5390 (16)	C12—C13	1.401 (2)
C2—C9	1.5801 (16)	C12—H12	0.9500
C2—H2	1.0000	C13—C14	1.401 (2)
C3—O3	1.2180 (15)	C13—H13	0.9500
C3—C4	1.5279 (18)	C14—C15	1.3923 (17)
C4—C5	1.5368 (15)	C14—H14	0.9500
C4—H4A	0.9900	C15—H15	0.9500
C4—H4B	0.9900	C16—C17	1.5329 (16)
C5—N8	1.5082 (14)	C16—H16A	0.9900
C5—C6	1.5742 (18)	C16—H16B	0.9900
C5—H5	1.0000	C17—C22	1.4091 (19)
C6—C7	1.5762 (18)	C17—C18	1.4140 (19)
C6—H6A	0.9900	C18—C19	1.4116 (18)
C6—H6B	0.9900	C18—H18	0.9500
C7—H7A	0.9900	C19—C20	1.406 (2)
C7—H7B	0.9900	C19—H19	0.9500
N8—C16	1.4861 (14)	C20—C21	1.403 (2)
O9—C9	1.4430 (16)	C20—H20	0.9500
O9—H9	0.8400	C21—C22	1.4151 (19)
C9—C10	1.5121 (16)	C21—H21	0.9500
C9—H9C	1.0000	C22—H22	0.9500
			
N8—C1—C2	107.58 (10)	O9—C9—H9C	107.8
N8—C1—C7	105.46 (9)	C10—C9—H9C	107.8
C2—C1—C7	111.25 (10)	C2—C9—H9C	107.8
N8—C1—H1	110.8	C15—C10—C11	120.07 (11)
C2—C1—H1	110.8	C15—C10—C9	121.20 (12)
C7—C1—H1	110.8	C11—C10—C9	118.73 (12)
C3—C2—C1	108.74 (9)	C12—C11—C10	120.33 (13)
C3—C2—C9	112.33 (10)	C12—C11—H11	119.8
C1—C2—C9	111.67 (9)	C10—C11—H11	119.8
C3—C2—H2	108.0	C11—C12—C13	119.32 (13)
C1—C2—H2	108.0	C11—C12—H12	120.3
C9—C2—H2	108.0	C13—C12—H12	120.3
O3—C3—C4	121.66 (11)	C12—C13—C14	120.62 (11)
O3—C3—C2	121.38 (12)	C12—C13—H13	119.7
C4—C3—C2	116.94 (10)	C14—C13—H13	119.7
C3—C4—C5	109.85 (10)	C15—C14—C13	120.37 (13)
C3—C4—H4A	109.7	C15—C14—H14	119.8
C5—C4—H4A	109.7	C13—C14—H14	119.8
C3—C4—H4B	109.7	C14—C15—C10	119.27 (13)
C5—C4—H4B	109.7	C14—C15—H15	120.4
H4A—C4—H4B	108.2	C10—C15—H15	120.4
N8—C5—C4	108.25 (9)	N8—C16—C17	111.62 (9)
N8—C5—C6	105.38 (10)	N8—C16—H16A	109.3
C4—C5—C6	109.80 (10)	C17—C16—H16A	109.3
N8—C5—H5	111.1	N8—C16—H16B	109.3
C4—C5—H5	111.1	C17—C16—H16B	109.3
C6—C5—H5	111.1	H16A—C16—H16B	108.0
C5—C6—C7	103.74 (10)	C22—C17—C18	118.94 (12)
C5—C6—H6A	111.0	C22—C17—C16	120.10 (12)
C7—C6—H6A	111.0	C18—C17—C16	120.96 (12)
C5—C6—H6B	111.0	C19—C18—C17	120.96 (13)
C7—C6—H6B	111.0	C19—C18—H18	119.5
H6A—C6—H6B	109.0	C17—C18—H18	119.5
C1—C7—C6	104.36 (10)	C20—C19—C18	119.68 (13)
C1—C7—H7A	110.9	C20—C19—H19	120.2
C6—C7—H7A	110.9	C18—C19—H19	120.2
C1—C7—H7B	110.9	C21—C20—C19	119.74 (12)
C6—C7—H7B	110.9	C21—C20—H20	120.1
H7A—C7—H7B	108.9	C19—C20—H20	120.1
C16—N8—C1	112.14 (10)	C20—C21—C22	120.66 (13)
C16—N8—C5	109.34 (9)	C20—C21—H21	119.7
C1—N8—C5	101.69 (9)	C22—C21—H21	119.7
C9—O9—H9	109.5	C17—C22—C21	120.02 (13)
O9—C9—C10	109.26 (11)	C17—C22—H22	120.0
O9—C9—C2	112.65 (9)	C21—C22—H22	120.0
C10—C9—C2	111.47 (10)		

### Hydrogen-bond geometry (Å, °)

**Table:**

*D*—H···*A*	*D*—H	H···*A*	*D*···*A*	*D*—H···*A*
O9—H9···N8	0.84	1.99	2.7280 (13)	146
C6—H6B···O3^i^	0.99	2.61	3.3414 (15)	131
C7—H7A···O3^i^	0.99	2.52	3.2954 (15)	135
C16—H16A···O3^i^	0.99	2.60	3.5846 (16)	173

Symmetry codes: (i) −*x*+2, *y*+1/2, −*z*+1/2.
